# Non-exanthematous mpox and the implications on response measures during outbreaks

**DOI:** 10.11604/pamj.supp.2025.50.1.49918

**Published:** 2025-12-01

**Authors:** Allan Komakech, Dativa Aliddeki

**Affiliations:** 1 School of Public Health, Clarke International University, Kampala, Uganda,; 2 Uganda National Institute of Public Health, Ministry of Health, Kampala, Uganda,; 3 School of Global Health and Ethical Studies, EUCLID (Euclid University/Pôle Universitaire Euclide), Bangui, Central African Republic,; 4 Africa Centres for Disease Control and Prevention (Africa CDC), Nairobi, Kenya

**Keywords:** Non-exanthematous, mpox, implications, public health, outbreaks

## Abstract

In 2024, mpox was declared a public health emergency. Several response measures have since been implemented by health authorities in the affected countries to control the outbreak. Our perspective was triggered by an article in the special issue on the surge of mpox in Africa that highlighted rashless or non-exanthematous presentations of mpox in Nigeria. Although these have been previously highlighted, especially during the global outbreak in 2022, the contribution of these factors to the general outbreak dynamics remains poorly understood. We reflect on the possible implications of this outbreak and strategies to address it in the context of a newer clade 1b responsible for the current upsurge, where no prevalence studies of rashless mpox have been done. Using our experiences, we also discuss how diagnostic expectations centred on the presence of rash may hinder timely detection, response, and reporting. Moreover, current outbreak situation reports do not disaggregate cases by symptom profile (for example, rash-presenting versus rashless), a gap that undermines a comprehensive understanding of the actual burden of mpox. Finally, we analyse the potential implications of undetected spread, surveillance, vaccination strategies and risk communication and behavioural change practices, to help break the chains of transmission that might have been missed.

## Perspective

In August 2024, the Africa Centres for Disease Control (Africa CDC) and the World Health Organization (WHO) both escalated mpox to emergency status; Africa CDC declared it a Public Health Emergency of Continental Security, and WHO declared it a Public Health Emergency of International Concern [1,2]. Following these declarations, the technical advisory groups of both organisations regularly reviewed this status based on ongoing transmission and response challenges. However, in September 2025, WHO declared the end of the mpox outbreak as an emergency of international concern [3]. As of the end of 19 October 2025, there were 121,151 suspected human cases and 735 deaths (case fatality rate of 0.61%) in 2025 across 28 African countries [4]. The large majority of these countries were grappling with the newer clade 1b variant of the virus, which was discovered in the Democratic Republic of Congo in late 2023 [5].

**The trigger:** reading an article in this special issue on the surge of mpox on the African continent, which referenced non-exanthematous mpox presentations in Nigeria, immediately caught our attention [6]. It validated a lingering concern that we may be overlooking a subset of mpox cases simply because they do not present with the classic rash. These rashless presentations could be contributing to ongoing transmission while escaping routine detection. Although we have not encountered a confirmed mpox case without a rash in our own field practice, the idea is not far-fetched. Discussions with colleagues and anecdotal reports from outbreak zones suggest that such cases may exist and go undiagnosed. The mere possibility warrants serious consideration and action.

**Prevalence of non-exanthematous mpox:** before the multicountry mpox outbreak in 2022-2023, primarily caused by clade IIb, mpox was mostly regarded as a symptomatic disease, almost always presenting with a characteristic rash, fever, and lymphadenopathy, all of which are considered pathognomonic symptoms. However, recent studies have documented infected individuals who do not develop the typical exanthem. A study conducted in Madrid, Spain, in 2022, during the global mpox outbreak, highlighted the presence of mpox among individuals who lacked the classical exanthem (2 cases, 1.8%), indicating the possibility of transmission [7]. A large case series study in 16 countries similarly highlighted a prevalence of 5% rashless mpox among 528 cases [8]. Similarly, 10% of the cases from this multicountry study also presented with single lesions in the genital areas, creating a possibility of missed diagnosis, as this may also be considered rashless or confused with other disease conditions [8]. This study that triggered our attention identified two individuals (2.67%) who presented with fever, lymphadenopathy, mucosal ulcers and proctitis but with no cutaneous lesions. These emerging data suggest that a rash is not an obligatory sign of mpox, and symptomatic illness can exist even without a rash. Importantly, patients with non-exanthematous symptoms such as isolated proctitis, dysuria, mucosal ulcers, or unexplained fever may be misdiagnosed or not tested for mpox at all under the current case definitions. Our discussions with clinicians indicate that some cases were only identified retrospectively, often after transmission had already occurred. Equally important is the fact that, to date, we have not found any situation report or official surveillance summary that analyses what proportion of mpox patients lacked a rash. This omission means we are likely underestimating the disease´s burden and the extent of undetected transmission, as well as the implications for outbreak control. It is also important to note that no comprehensive prevalence studies have been conducted in Africa to document non-exanthematous mpox, especially during the multicountry outbreak declared in 2024-2025, when the predominant clade 1b was identified.

### Public health implications and recommendations

**Rethinking undetected spread:** when some mpox infections are undetected, it may be impossible to identify all the transmission chains of the disease, even while isolating the rash-presenting cases and their contacts. Undiagnosed carriers could unknowingly continue to fuel an outbreak. A modelling study in the 2022 outbreak suggests that the actual number of mpox might have been underestimated by several fold, with this attributed in part to undetected mild or atypical cases, including non?exanthematous presentations [9]. In high-risk environments such as sexual health contexts, partners may fail to avoid contact with someone who does not have visible lesions, due to the assumption that there is no risk. This implies that in known epidemic areas, and in high-risk environments, testing should be done widely with an increased index of suspicion, even including high-risk contacts, to identify some mpox infections which may be rashless or atypically presenting mpox. However, understanding the role of these rashless and atypical mpox cases is ongoing work, with a call that has been presented by other researchers previously [10].

**Enhanced surveillance:** there have been calls for broader surveillance and case-finding efforts, particularly in high-risk areas and high-risk populations, to better capture atypical and rashless cases. These efforts require clinicians to increase their index of suspicion, especially in contexts of epidemiological risk. They should be able to identify rashless lesions in the presence of other symptoms, as well as in single-lesion presentations. In low-resource settings, this intervention may not be feasible or practical, making the case for frequent risk mapping to inform interventions. Countries such as Spain have previously conducted proactive screening programs, showing their feasibility and usefulness. In Spain, a self-sampling initiative also helped identify infections among men at high risk, infections that would have been missed by symptom-based testing alone. Expanding surveillance to include high-risk individuals with atypical symptoms (such as mucosal lesions, rectal pain or proctitis, urinary symptoms or unexplained fever) would help uncover hidden chains of transmission. National surveillance programmes should be designed to detect non-exanthematous mpox cases, making mpox testing more accessible (e.g., via rapid tests or mail-in kits) and integrating mpox screening into routine sexual health check-ups and in HIV settings. If we rely solely on individuals to self-identify based on having an exanthematous rash or known exposure, we will miss a subset of mpox cases.

**Vaccination strategies:** traditionally, the ring vaccination approach, where contacts of infected persons are vaccinated first, was considered the most potent approach. However, the possibility of having non?exanthematous mpox infections has implications for the vaccination policy and strengthens the case for pre-exposure vaccination in high-risk groups. This is particularly useful if indeed those who do not have the classical rash can transmit the infection to others. The uptake of pre-exposure vaccination was particularly notable during the 2022-2023 global outbreak, even without known contacts. This approach has also been adopted in Africa during the more recent multicountry outbreak, a key recommendation from the Africa CDC and the WHO [11]. Widely vaccinating vulnerable populations, coupled with community engagement, can therefore be a key long-term preventive effort. Recent data indicate that two-dose vaccination provides substantial protection against mpox infection [12]. Achieving high coverage in high-risk populations and areas of high transmission would significantly mitigate the chance of silent spread. However, countries should then focus on addressing the recurrent challenge of vaccine supply and the use of a microtargeted deployment strategy. Vaccination efforts have also faced the challenge of reaching some vulnerable populations without stigmatising them. These challenges should be addressed to counter the silent spread of infections.

**Risk communication and behavioural change:** a cornerstone in reducing the risk of silent infection spread through non-classical rashes is effective public health communication. Messaging from health communicators must be adapted to acknowledge that mpox infection isn´t always obvious. Campaigns should convey that if an individual has a high-risk exposure, even without symptoms, or develops atypical symptoms such as flu-like symptoms (fever, headache) or other symptoms like mucosal ulcers, rectal pain, or urinary discomfort, the possibility of having mpox infection should be considered. These cases should warrant testing for mpox, even in the absence of a rash, especially when sufficient diagnostic tools are available, as already emphasised by the WHO. Health messaging can encourage individuals to be vigilant for any signs of illness, no matter how mild or atypical, after potential exposure. During outbreak peaks, temporary behaviour modifications, such as reducing one´s number of sexual partners or avoiding anonymous sexual encounters, may be prudent because not all infectious cases will be visibly sick [13]. However, communication should still be rights-based and confidentiality assured, as this may depress care-seeking and skew surveillance, as noticed in Nigeria [14]. Additionally, continuous reiteration and reinforcement of general preventive practices remain essential. Current WHO and national guidelines still emphasise the need to isolate until completely healed among those with symptoms, as the primary measure to prevent the spread of infection. However, the possibility of non?exanthematous mpox adds more importance to other measures, such as consistent condom use. It is also crucial to inform frontline health workers about the evolving clinical picture of mpox to maintain a high index of suspicion, particularly among high-risk populations and in areas with high transmission, even in the absence of a rash.

## Conclusion

We write this perspective because we believe our current and past responses to mpox may have a missing link, particularly for the clade 1b outbreak in Africa. Previously, a focus on the classical presentation with an exanthematous rash may have led to unidentified transmission chains. Recognising such infections requires efforts to curb the undetected spread, such as revising testing strategies guided by frequent risk assessments and inclusive surveillance, especially for high-risk populations and areas with high transmission rates. Restrategising vaccination approaches for those at risk before exposure and refining communication strategies to highlight atypical mpox presentations are vital interventions. Ultimately, we must adapt our epidemiological thinking and mindset to reflect realities on the ground and strive to identify all mpox transmission links.
